# Sural Nerve Entrapment in Postoperative Scar Tissue: A Case of Successful Management With Hydrodissection After Calcaneal Fracture Repair

**DOI:** 10.7759/cureus.96069

**Published:** 2025-11-04

**Authors:** Yonghyun Yoon, King Hei Stanley Lam, Ji Hyo Hwang, Dongyeun Sung, Jaeyoung Lee, Teinny Suryadi, Daniel Chiung-Jui Su

**Affiliations:** 1 Orthopaedic Surgery, Hallym University Kangnam Sacred Heart Hospital, Seoul, KOR; 2 Orthopaedics, Incheon Terminal Orthopedic Surgery Clinic, Incheon, KOR; 3 Clinical Research, International Academy of Regenerative Medicine, Incheon, KOR; 4 Clinical Research, International Academy of Musculoskeletal Medicine, Kowloon, HKG; 5 Faculty of Medicine, The University of Hong Kong, Hong Kong, HKG; 6 Faculty of Medicine, The Chinese University of Hong Kong, New Territories, HKG; 7 Clinical Research, The Hong Kong Institute of Musculoskeletal Medicine, Kowloon, HKG; 8 Neurosurgery, Himchannamu Neurosurgery Clinic, Daegu, KOR; 9 Physical Medicine and Rehabilitation, Synergy Clinic, Jakarta, IDN; 10 Physical Medicine and Rehabilitation, Hermina Podomoro Hospital, Jakarta, IDN; 11 Physical Medicine and Rehabilitation, Chi Mei Medical Center, Tainan, TWN

**Keywords:** calcaneus fracture, hydrodissection, nerve entrapment, neuropathic pain treatment, post-surgical neuropathy, post-surgical pain, post surgical scar, sonoguided digital palpation, sural nerve, ultrasound-guided intervention

## Abstract

Sural nerve entrapment is a recognized yet frequently overlooked etiology of persistent lateral ankle and heel pain following lower extremity trauma or surgery. This report describes a case of refractory sural nerve entrapment within scar tissue that persisted after calcaneal fracture repair and subsequent hardware removal, which was successfully managed with ultrasound-guided hydrodissection.

A 31-year-old male presented with refractory right heel pain following open reduction and internal fixation (ORIF) for a calcaneal fracture and subsequent elective hardware removal. Physical examination revealed allodynia and hyperesthesia along the surgical scar, accompanied by a positive jump sign. Diagnostic ultrasonography confirmed entrapment of the sural nerve within adjacent hypoechoic scar tissue. The diagnosis was dynamically confirmed using sonoguided digital palpation (SDP), which precisely reproduced the patient’s characteristic pain at the site of nerve-scar adhesion. The patient subsequently underwent two sessions of ultrasound-guided hydrodissection with 5% dextrose in water (D5W). He experienced significant and immediate pain relief after the first procedure, reporting over 90% symptom resolution following the second session, which was sustained at follow-up.

This case highlights that sural nerve entrapment should be considered in the differential diagnosis of persistent lateral heel pain following calcaneal surgery, even after hardware removal. A comprehensive diagnostic workup incorporating dynamic ultrasound with SDP can reliably identify and confirm the symptomatic site of entrapment. Ultrasound-guided hydrodissection represents a safe, effective, and minimally invasive therapeutic option that can facilitate substantial symptom relief and functional improvement, potentially obviating the need for more extensive surgical interventions.

## Introduction

The sural nerve is a purely sensory nerve derived from the L4-S1 nerve roots. It typically forms in the distal third of the leg from the union of the medial sural cutaneous nerve (a branch of the tibial nerve) and the lateral sural cutaneous nerve (a branch of the common peroneal nerve) [1]. The nerve courses superficially alongside the small saphenous vein, passing posterior to the lateral malleolus to provide sensation to the posterolateral aspect of the leg and the lateral foot. This superficial anatomical trajectory renders it particularly vulnerable to external compression, trauma, and iatrogenic injury during surgical procedures such as calcaneal open reduction and internal fixation (ORIF), varicose vein surgery, or tendon repair.

Persistent pain following a calcaneal fracture is most frequently attributed to sequelae such as subtalar joint arthritis, malunion, or residual bony prominences [2-4]. Consequently, standard diagnostic and therapeutic approaches often focus on these articular and osseous abnormalities. However, neuropathic pain originating from nerve entrapment requires a distinctly different management strategy and is often overlooked. A recent report described sural nerve-related pain secondary to direct hardware irritation, where symptoms improved temporarily with hydrodissection but recurred, achieving complete resolution only after subsequent hardware removal [5].

In contrast to this previously described mechanism, the present case report describes a unique scenario of sural nerve entrapment within post-surgical scar tissue that persisted despite elective hardware removal. This case underscores that neuropathic pain from scar entrapment can be the sole cause of refractory symptoms even after the removal of hardware. We document the successful management of this condition using ultrasound-guided hydrodissection, thereby highlighting its therapeutic potential as a definitive treatment for post-surgical neuropathic pain refractory to standard surgical interventions.

## Case presentation

A 31-year-old male presented in March 2025 with refractory right heel pain following ORIF via an extended lateral approach for a calcaneal fracture in October 2021, followed by elective hardware removal 18 months later (Table 1). He had no significant past medical history and was not taking any medications.

**Table 1:** Clinical timeline from initial fracture repair to final follow-up. This table outlines the chronological sequence of key clinical events for the patient, from the initial surgical repair of the calcaneal fracture to the final assessment following hydrodissection therapy. The timeline emphasizes the persistence of symptoms for a significant period after hardware removal, culminating in the presentation with refractory pain and subsequent intervention. ORIF: Open reduction and internal fixation.

Event	Date / Timeframe
Initial calcaneal ORIF	October 2021
Elective hardware removal	Approximately 18 months post-ORIF
Presentation for refractory pain	March 2025
First hydrodissection session	March 2025
Second hydrodissection session	One week after first session
Final follow-up assessment	Six months after initial presentation

**Figure 1:**
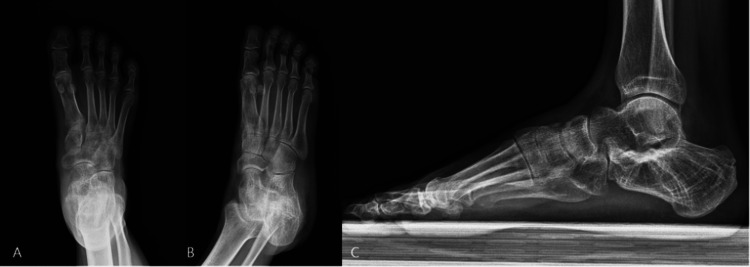
Radiographic evaluation of foot pathologies. Foot X-ray series: (A) anteroposterior (AP) view; (B, C) lateral views demonstrating mild osteoarthritis at the calcaneocuboid and subtalar joints.

The patient’s primary complaint was sharp pain upon heel strike during gait. At initial presentation, he rated his pain as 9/10 on the Visual Analog Scale (VAS). Observational gait analysis revealed an antalgic pattern with avoidance of the lateral aspect of the foot during weight-bearing. The surgical scar from the extended lateral approach was well healed. Compared with the contralateral side, allodynia and hyperesthesia were evident in the distribution of the sural nerve distal to the scar. Plain radiographs showed mild subtalar joint osteoarthritis but no evidence of a residual lateral wall fracture fragment or exostosis (Figure 1).

**Figure 2:**
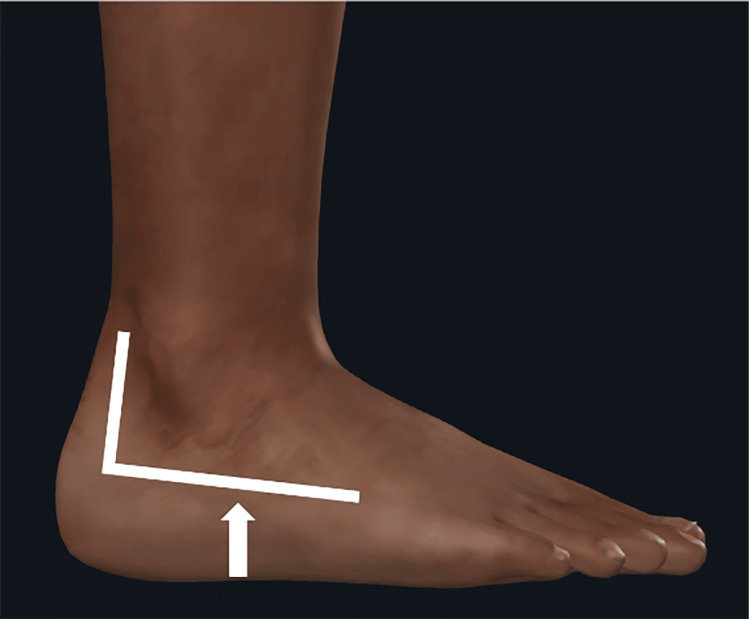
Clinical assessment of the lateral ankle. Lateral ankle surgical scar. The white arrow indicates the point of maximal tenderness corresponding to the positive jump sign.

Palpation of the surgical scar elicited a positive jump sign (a sudden, involuntary withdrawal response) at its distal portion, which corresponded to the area of allodynia and hyperesthesia (Figure 2).

Diagnostic USG demonstrated compression of the sural nerve by adjacent hypoechoic scar tissue, although its continuity remained intact (Video 1).

**Video 1:** Ultrasonographic evaluation of sural nerve compression. Ultrasonography demonstrating compression of the sural nerve by adjacent hypoechoic scar tissue.

**Figure 3:**
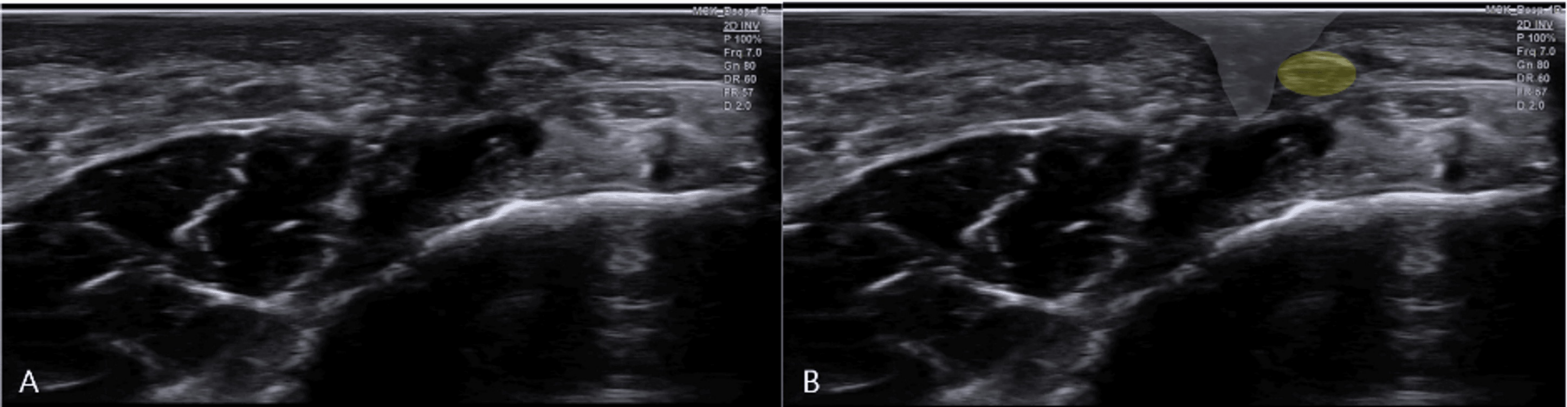
Ultrasonographic imaging of sural nerve compression. Ultrasonographic images of the symptomatic area: (A) B-mode image; (B) the sural nerve (yellow outline) compressed by surrounding scar tissue (grey outline).

Critically, SDP was performed, during which direct ultrasound-guided pressure over the site of nerve-scar adhesion precisely reproduced the patient’s characteristic neuropathic pain. This maneuver confirmed the exact site of entrapment as the source of symptoms (Figure 3, Video 2).

**Video 2:** Sonoguided digital palpation for nerve-scar adhesion assessment. Sonoguided digital palpation (SDP) demonstrating direct ultrasound-guided pressure applied to the site of nerve-scar adhesion. This maneuver precisely reproduces the patient’s characteristic neuropathic pain, confirming the exact site of entrapment as the source of symptoms.

**Figure 4:**
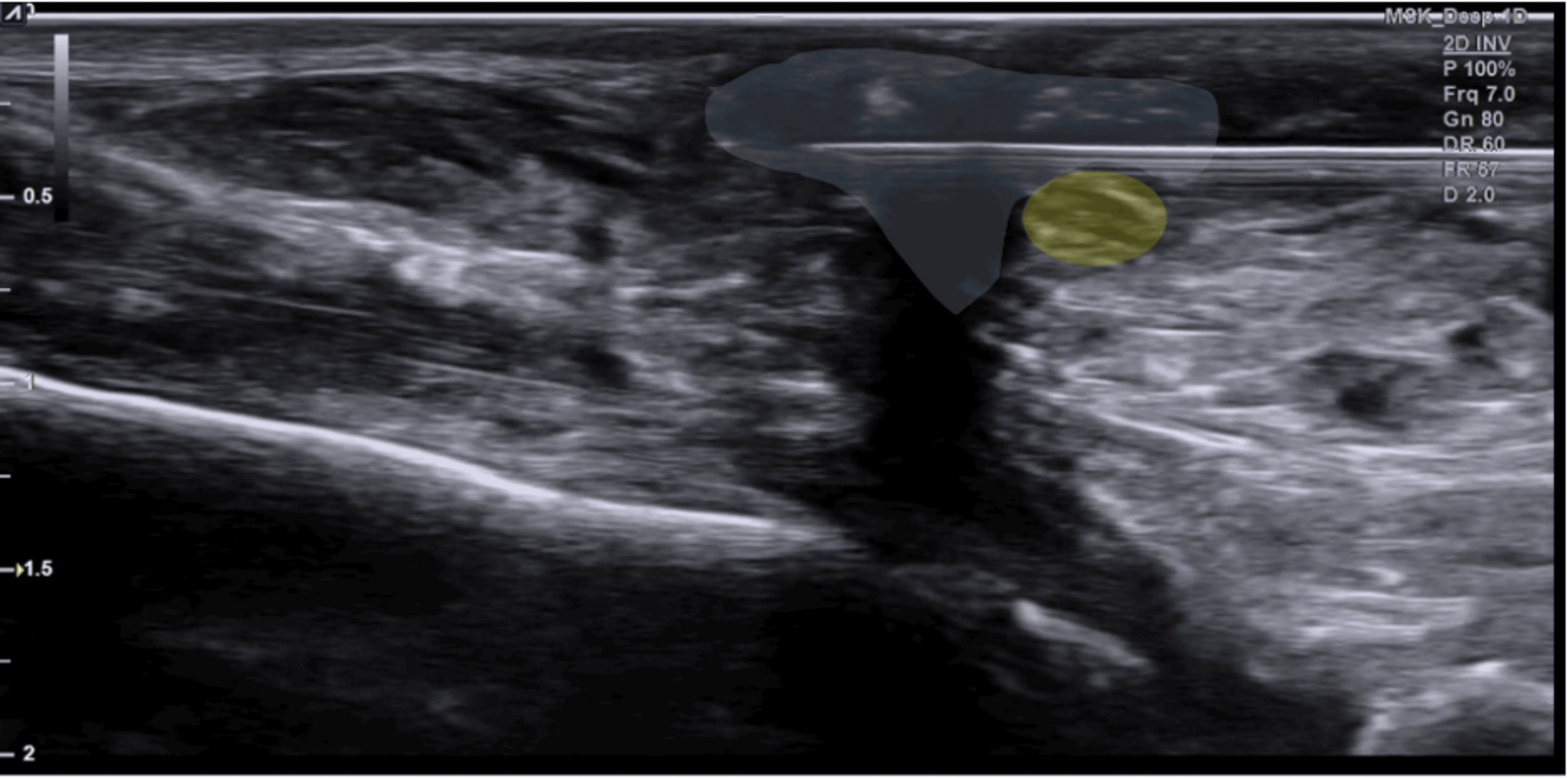
Ultrasonographic imaging of the hydrodissection procedure. Ultrasonographic image obtained during hydrodissection. The injected fluid (anechoic area) creates a separation plane between the sural nerve and the surrounding scar tissue.

Based on these findings, a diagnosis of sural nerve entrapment within post-surgical scar tissue was confirmed. The patient subsequently underwent ultrasound-guided hydrodissection with 20 mL of 5% dextrose in water (D5W) using a 25-gauge needle to mechanically separate the nerve from the adherent scar (Figure 4, Video 3).

**Video 3:** Ultrasound-guided hydrodissection of the sural nerve. Ultrasound-guided hydrodissection of the sural nerve. The needle tip (not shown) injects fluid, creating an anechoic area that forms a separation plane between the sural nerve and the surrounding scar tissue.

Immediately following the procedure, the patient reported a significant reduction in allodynia and hyperesthesia. His VAS score improved from 9/10 to 5/10, and his gait pattern also improved, with markedly decreased pain upon heel strike. The patient received a second hydrodissection session one week later. Following the second session, his pain further decreased to a VAS score of 1/10. At follow-up assessments three and six months later, he reported sustained symptom resolution with a stable VAS score of 1/10, representing over 90% improvement from his initial presentation.

## Discussion

This report demonstrates that sural nerve entrapment within post-surgical scar tissue is a diagnosable and treatable cause of refractory lateral heel pain following calcaneal ORIF. Critically, our findings indicate that this neuropathic pain syndrome can persist even after hardware removal and can be successfully resolved with targeted, minimally invasive intervention, thereby obviating the need for major salvage surgery.

This case illustrates a less common but important sequela of calcaneal fracture surgery. While post-traumatic subtalar arthritis is a well-recognized cause of persistent pain [2-4], our experience underscores the necessity of including peripheral nerve entrapment in the differential diagnosis, especially when pain is neuropathic in character, manifesting as allodynia and hyperesthesia, and localized to a specific nerve’s distribution.

The management of chronic pain following calcaneal fracture remains challenging [2,4]. When conservative measures fail and pain is attributed to the subtalar joint, triple arthrodesis is often considered the gold-standard surgical intervention. However, outcomes are not always satisfactory, and persistent pain after arthrodesis has been reported [6]. In such scenarios, an undiagnosed nerve entrapment could be the underlying culprit. Therefore, in patients with persistent pain even after hardware removal, peripheral nerve entrapment should be systematically evaluated before proceeding with major reconstructive procedures such as triple arthrodesis.

The diagnostic challenge can be understood through Hilton’s law [7], which posits that the nerves innervating a joint also supply the muscles acting on it and the skin overlying it. Given that the ankle and subtalar joints are innervated by branches of the tibial, superficial peroneal, deep peroneal, and sural nerves, pain may originate not only from the joint itself but also from injured or entrapped nerves in the vicinity. Therefore, in cases of chronic post-surgical foot and ankle pain, a targeted diagnostic workup for nerve entrapment is warranted before proceeding with major salvage surgery such as arthrodesis.

Ultrasound-guided hydrodissection has emerged as a potent, minimally invasive technique for treating nerve entrapments [8,9]. The procedure involves injecting a fluid medium to create a plane between the nerve and the surrounding adhesions or fibrotic tissue, thereby freeing the nerve and restoring its gliding function. The use of D5W, an iso-osmolar solution, may provide additional therapeutic benefits by reducing neurogenic inflammation and acting as a mild neurolytic agent to desensitize nerves, although its exact mechanism is still being elucidated [8,9]. The significant and sustained pain relief, as quantified by the VAS scores, experienced by our patient highlights the efficacy of this intervention.

The key to successful management lies in a meticulous diagnostic process combining a detailed history, a physical examination with targeted provocative maneuvers (e.g., the jump sign), and high-resolution USG for dynamic confirmation. In our case, the use of SDP was instrumental [10]. This technique allows the clinician to correlate the anatomical abnormality observed on ultrasound, the nerve tethered by scar tissue, with the patient’s live pain response, providing a dynamic and objective confirmation of the symptomatic entrapment site before initiating therapy [8,9].

Our findings are consistent with the growing body of evidence supporting hydrodissection for peripheral nerve entrapment and specifically corroborate its utility in managing sural neuropathy following calcaneal surgery, as recently reported by Omodani T and Takahashi K (Table 2) [5]. However, critical differences between the cases highlight important clinical nuances. In the case described by Omodani T and Takahashi K, hydrodissection provided significant but incomplete relief, and the patient ultimately required hardware removal and surgical neurolysis for full resolution. This suggests that when a prominent mechanical irritant, such as a fixation plate, is present, hydrodissection may serve as a valuable bridging or diagnostic therapy but not always a definitive one. Our case presents a distinct and important scenario in which nerve entrapment persisted solely due to scar tissue, as the hardware had already been removed. Furthermore, the definitive diagnostic confidence provided by dynamic SDP in our case may have contributed to the precise targeting of the hydrodissection. The fact that two sessions of hydrodissection with D5W alone resulted in sustained and near-complete symptom resolution (VAS 9/10 → 1/10) demonstrates that for pure fibrotic entrapment, hydrodissection can serve as a curative, standalone intervention. This distinction underscores the importance of using advanced ultrasound techniques to precisely identify the etiology of entrapment, as the treatment pathway and prognosis may differ significantly.

**Table 2:** Comparative analysis of sural nerve entrapment cases following calcaneal fracture repair. This table presents a comparison between the current case and a previously reported instance of sural neuropathy following calcaneal fracture surgery. Notable differences highlight how the underlying cause of nerve entrapment, whether due to hardware or isolated scar tissue, significantly influences treatment strategies and outcomes. While hydrodissection served as a temporary measure in cases involving hardware, it proved to be a definitive and curative approach for entrapment caused solely by post-surgical fibrosis, even when prior hardware removal had already been performed. Additionally, the choice of injectate may play a crucial role in determining the durability of treatment efficacy. Source: Reference [5].

Feature	Omodani T and Takahashi K (2023) Case	Current Case
Primary cause of entrapment	Hardware-related, pain was likely due to a combination of scar tissue and the physical presence of the fixation plate.	Scar tissue alone, entrapment persisted even after elective hardware removal, isolating the cause to post-surgical fibrosis.
Intervention	Hydrodissection (with 0.09% lidocaine) provided significant but temporary relief.	Hydrodissection (with 5% dextrose in water (D5W)) provided definitive, sustained relief after two sessions.
Ultimate resolution	Required subsequent surgery (plate removal and neurolysis) for complete symptom resolution.	Achieved with hydrodissection alone , no further surgery was required.
Injectate	0.09% lidocaine (a short-acting local anesthetic).	5% dextrose in water (D5W) without local anesthetic , a potentially therapeutic, mild neurolytic agent.
Clinical implication	Hydrodissection can serve as a useful bridging or diagnostic therapy in complex cases with hardware but may not be definitive.	Hydrodissection can be a curative, standalone treatment for pure scar tissue entrapment, even in refractory cases.
Key message	Hydrodissection is a valuable tool, but hardware removal may still be necessary for complete resolution.	For isolated sural nerve entrapment within scar tissue, hydrodissection can obviate the need for repeat surgery.

The definitive success of hydrodissection as a standalone treatment in our case, contrasting with the temporary relief described in other reports, may be attributed to several factors. First, the primary etiology in our patient was isolated fibrotic entrapment, as the hardware, a potential persistent mechanical irritant, had already been removed. This created a scenario in which mechanical liberation of the nerve was fully therapeutic. Second, our injectate of choice, D5W, may have provided benefits beyond the mechanical hydrodissection achieved with local anesthetic. While lidocaine primarily offers temporary analgesia, D5W is hypothesized to exert prolonged effects on neurogenic inflammation and sensitized C-fibers, potentially acting as a mild neurolytic agent that provides longer-lasting desensitization of the irritated nerve [8,9]. Finally, the administration of a second hydrodissection session one week after the first may have reinforced both the initial mechanical release and the anti-inflammatory effect, ensuring more durable separation of the nerve from reforming scar tissue.

Limitations

Several limitations should be considered when interpreting the findings of this case report. The most significant is its design as a single case report, which, while illustrative, limits the generalizability of the results. Therefore, the efficacy and safety of hydrodissection for this specific condition require validation in larger, controlled studies.

Second, the diagnosis was based on a combination of physical examination and dynamic USG with SDP, without the use of a preceding diagnostic nerve block. Although a diagnostic block can provide independent confirmatory evidence, the patient’s pronounced jump sign, clear sonographic evidence of entrapment, and precise pain reproduction during SDP established a compelling diagnostic chain, further supported by the immediate and sustained therapeutic response and the objective improvement in VAS scores.

Lastly, the follow-up period in this case is of medium duration. Longer-term monitoring is necessary to confirm that the symptom relief achieved through hydrodissection is durable and not subject to recurrence as scar tissue potentially reforms.

## Conclusions

This report highlights that sural nerve entrapment is a diagnostically tractable cause of refractory lateral heel pain following calcaneal surgery, which can persist even after hardware removal. A comprehensive evaluation incorporating dynamic ultrasonography supplemented by SDP can reliably identify and confirm the symptomatic site of nerve entrapment. Ultrasound-guided hydrodissection with D5W represents a safe, effective, and minimally invasive therapeutic option that can lead to significant and sustained symptom resolution with improved functional outcomes. This intervention should be considered a primary treatment strategy for suspected post-surgical nerve entrapment before proceeding with more extensive salvage procedures. Consequently, we propose that in the diagnostic algorithm for persistent pain following calcaneal ORIF, particularly when hardware removal has failed to provide relief, sural nerve entrapment must be definitively ruled out using ultrasound-guided assessment before considering major orthopedic salvage procedures such as triple arthrodesis.
